# A Comparative Study on Force-Fields for Interstitial Diffusion in α-Zr and Zr Alloys

**DOI:** 10.3390/ma17153634

**Published:** 2024-07-23

**Authors:** Jing Li, Tan Shi, Chen Zhang, Ping Zhang, Shehu Adam Ibrahim, Zhipeng Sun, Yuanming Li, Chuanbao Tang, Qing Peng, Chenyang Lu

**Affiliations:** 1 Department of Nuclear Science and Technology, Xi’an Jiaotong University, Xi’an 710049, China; 4120103221@stu.xjtu.edu.cn (J.L.); tan.shi0122@xjtu.edu.cn (T.S.); 3123103370@stu.xjtu.edu.cn (C.Z.); zp719813@stu.xjtu.edu.cn (P.Z.); shehu.adam@yahoo.com (S.A.I.); chenylu@xjtu.edu.cn (C.L.); 2 Nuclear Power Institute of China, Chengdu 610213, China; superszp@163.com (Z.S.); tcb_npic@163.com (C.T.); 3 State Key Laboratory of Nonlinear Mechanics, Institute of Mechanics, Chinese Academy of Sciences, Beijing 100190, China; 4 School of Engineering Sciences, University of Chinese Academy of Sciences, Beijing 100049, China; 5 Guangdong Aerospace Research Academy, Guangzhou 511458, China; 6 State Key Laboratory of Multiphase Flow in Power Engineering, Xi’an Jiaotong University, Xi’an 710049, China

**Keywords:** zirconium alloys, interstitial diffusion, radiation damage, interatomic potentials, molecular dynamics

## Abstract

Interstitial diffusion is important for radiation defect evolution in zirconium alloys. This study employed molecular dynamics simulations to investigate interstitial diffusion in α-Zr and its alloys with 1.0 at.% Nb and 1.0 at.% Sn using a variety of interatomic potentials. Pronounced differences in diffusion anisotropy were observed in pure Zr among the employed potentials. This was attributed to the considerable differences in migration barriers among the various interstitial configurations. The introduction of small concentrations of Nb and Sn solute atoms was found to significantly influence diffusion anisotropy by either directly participating in the diffusion process or altering the chemical environment around the diffusing species. Based on the moderate agreement of interstitial energetics in pure Zr, accurately describing interstitial diffusion in Zr alloys is expected to be more complex. This work underscores the importance of the careful validation and selection of interatomic potentials and highlights the need to understand the effects of solute atoms on interstitial diffusion.

## 1. Introduction

Zirconium (Zr) alloys are commercial cladding materials widely used in nuclear reactors due to their low neutron absorption cross-section, excellent mechanical properties, and radiation and corrosion resistance [1,2,3]. There have been active research efforts [4,5] aimed at understanding Zr-Nb and Zr-Sn-Nb alloys’ in-reactor behavior and enhancing their material properties. When Zr alloys are subjected to neutron irradiation in a reactor environment, a significant amount of radiation defects are produced, leading to the potential degradation of their material properties [6]. Despite being a conventional nuclear material, understanding their irradiation damage mechanisms remains of great importance for the evaluation of their material properties in the context of accidental scenarios and reactor extension, as well as for the development of novel Zr alloys. Point defects and defect clusters, as basic types of radiation defects, play critical roles in the evolution of interstitial/vacancy-type dislocation loops and radiation growth [7,8,9]. Moreover, they also affect the migration of solute elements via vacancy or interstitial-mediated diffusion [10,11], further influencing the microstructure evolution and impacting the corrosion resistance [4,12,13].

Molecular dynamics (MD) simulations are a useful method for studying the evolution of radiation-induced defects in Zr alloys [14,15,16]. Based on the interatomic potentials of pure Zr and Zr alloys, the energetics of point defects and defect clusters and the interactions between Zr and solute elements can be studied from an atomistic perspective. Additionally, the obtained results can also be utilized as input parameters for modeling at larger scales, including cluster dynamics or rate theory [14,15]. In Zr and Zr alloys with a hexagonal closed packed (HCP) structure, an accurate description of the diffusion anisotropy is important for understanding the radiation defect evolution. It has been shown that the diffusion anisotropy of point defects and self-interstitial atom (SIA) clusters are mostly responsible for the growth of dislocation loops in different directions, including the coexistence of vacancy- and interstitial-type prismatic loops (a-loops) and the dominance of vacancy-type c-loops [7,8,9,16]. This leads to the anisotropic growth of Zr alloys with tensile deformation in the basal planes and compressive deformation along the c-direction [16]. It is noted that simulating SIA diffusion is not trivial, given that different SIA configurations or transition states can have very similar energies [1,9,17]. For Zr alloys, different solute elements also exhibit diffusion anisotropy and have differing diffusivities through vacancy-mediated or interstitial-mediated mechanisms [10,11], which influences solute segregation and precipitation. In this study, in order to assess the suitability of different interatomic potentials for defect diffusion anisotropy, molecular dynamics simulations were performed to compare the behavior of self-interstitial diffusion across various classical interatomic potentials and against previous density functional theory (DFT) calculations.

Various interatomic potentials for Zr and Zr alloys have been developed by researchers. Mendelev and Ackland et al. developed three EAM potentials for α-Zr. The third potential, referred to here as EAM-Mendelev#3 [18], is commonly used for irradiation damage simulations because it takes into account the formation energies of vacancy and various interstitial configurations. This early potential predicts the O configuration as the most stable SIA [18]. Similarly, the EAM potential for Zr developed by Ouyang et al. (denoted as EAM-Ouyang) also predicts a stable O-interstitial configuration [19]. However, DFT studies with large supercells revealed that BO is the most stable interstitial configuration and has a different migration mechanism compared with the O interstitial configuration [17,20]. The EAM potential function developed by Zhou et al. [21] (denoted as EAM-Zhou) provides a good prediction of the stable BO interstitial and suggests that the most common interstitial migration path in Zr is hopping between the two nearest basal octahedral (BO) sites on the basal plane. Moreover, the Zr-Nb angular-dependent potential (ADP) developed by Starikov et al. (denoted as ADP-Starikov) [22], which considers the solution energy and interstitial formation energy of the solution atoms, is used here to investigate Nb diffusion and its influence on Zr diffusion in a dilute Zr-Nb alloy. A similar analysis on interstitial diffusion was also conducted in this work for the dilute Zr-Sn alloy with the modified EAM (MEAM) potential and the machine learning moment tensor potential (MTP) developed by Mei et al. (denoted as MEAM-Mei and MTP-Mei) [23]. The interstitial diffusion of some of these potentials has also been previously studied by either the author themselves or other researchers [18,21,22,24]. For example, Zhou et al. investigated the diffusion behavior of radiation-induced point defects among Zr alloys by the means of the developed EAM potential, and pointed out that the SIAs exhibited obvious anisotropic diffusion characteristics at low temperatures (<600 K) [21]. The diffusion coefficients of Zr and Nb in α-Zr with point defects were investigated by Starikov et al. Their study indicated that the Nb atoms showed more anisotropy diffusion, and the diffusivity of the Nb atoms was lower than that of the Zr atoms in a Zr-Nb alloy [22]. It should be noted that these potentials include different physical, thermal, mechanical, and defect properties during the potential fitting process, focusing on different problems related to Zr or Zr alloys. Each potential also maintains its own balance among the accuracies of the considered properties. Our goal here is to present a comprehensive comparison among the widely used potentials and newly developed potentials, and evaluate these potentials in relation to the specific problems of interstitial diffusivity and anisotropy, without claiming the superiority of any particular potential.

In this work, using the aforementioned interatomic potentials, atomic and defect diffusion diffusivities were simulated at various temperatures along the basal plane and c-axis. The diffusion anisotropy between these two directions was also determined. Considering the alloy potentials, the influences of Nb and Sn solute atoms were investigated in dilute Zr alloys. Through the comparison of dynamic diffusion and static energies, it was shown that the energy of self-interstitial atoms in various stable and saddle configurations significantly influenced both the diffusion coefficient and diffusion anisotropy. Simulations of interstitial diffusion and static interstitial energy calculations in α-Zr and Zr alloys using various potentials revealed the complexity of accurately modeling their interstitial behavior. This highlights the need for validating and selecting suitable potentials and understanding the impact of alloying elements on interstitial diffusion.

## 2. Materials and Methods

The molecular dynamics simulations were performed with the large-scale Atomic/Molecular Massively Parallel Simulator (LAMMPS, lammps-3Mar2020) code [25]. The studied systems included pure α-Zr, α-Zr with 1.0 at.% Nb (denoted as Zr-1Nb), and α-Zr with 1.0 at.% Sn (Zr-1Sn), with potentials of EAM-Mendelev#3 [18], EAM-Ouyang [19], and EAM-Zhou [21] for pure Zr, ADP-Starikov potential [22] for pure Zr and Zr-1Nb, and MEAM-Mei and MTP-Mei potentials for pure Zr and Zr-1Sn [23], which were developed by Mendelev et al., Ouyang et al., Zhou et al., Starikov et al., and Mei et al. The simulation box consisted of 13,500 atoms with x, y, and z axes oriented along the [01$\bar{1}$0], [2$\bar{1}\bar{1}$0], and [0001] directions. A single interstitial was introduced into the system by adding a Zr atom. The diffusion was simulated using the canonical ensemble (NVT ensemble, with a constant number of atoms, temperature, and volume) [26] with a timestep of 1 fs for a total duration of at least 120 ns. The simulation time was extended to 180 ns for cases at low temperatures. Temperatures of 600 K, 800 K, 1000 K, 1200 K, and 1400 K were used. Owing to the slower computational speed of the machine learning MTP-Mei potential, only a single diffusion temperature was computed for comparison.

All the atoms were considered as tracers when the tracer diffusion coefficients ($D^{*}$) were calculated based on the atomic square displacement (ASD) [27]:

(1) $$D^{*}=\frac{1}{c_{d}}\times \frac{<R^{2}>}{2nt}$$

where <*R*^2^> is the average ASD, *c*_d_ is the defect concentration, *n* is the system dimensionality with *n* = 3 for total diffusion, *n* = 2 for basal diffusion, and *n* = 1 for c-axis diffusion, and *t* is the diffusion time. The Wigner–Seitz method [28] was used to identify the point defect positions, which were recorded every 20 ps, allowing the defect trajectories to be obtained. Then, the mean-square displacement (MSD) [29,30] of the point defect (denoted as <*r*^2^>) was calculated by the modified pymatgen program [31]. Compared to ASD, MSD takes an additional average over the time origins, allowing for a better statistical trend for a single defect trajectory [29,30]. The diffusion coefficient of the point defect ($D^{d}$) was then calculated by:

(2) $$D^{d}=\frac{<r^{2}>}{2nt}$$

Due to the large lattice vibration at high temperatures, additional Frenkel pairs could occasionally appear with the Wigner–Seitz method. In such instances, a comparison among the point defect positions allowed for the determination of the actual diffusing defect. The Arrhenius equation was used to determine the pre-exponential factor ($D_{0}$) and activation energy ($E_{a}$) for the tracer diffusion [27]:

(3) $$D=D_{0}exp(-\frac{E_{a}}{k_{b}T})$$

The diffusion anisotropy parameter was defined as the ratio of $D_{a}/D_{c}$, where $D_{a}$ and $D_{c}$ represent the basal plane and c-axis diffusion coefficients, and *k*_b_ is the Boltzmann constant.

**Figure 1:**
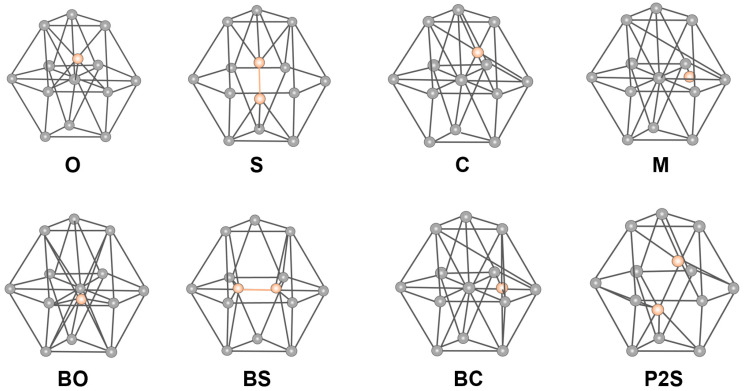
Different interstitial configurations of α-Zr. The gray spares mark the atoms in the lattice sites and the orange spheres mark the interstitial atoms.

In addition to the dynamic diffusion simulations, static calculations were performed to obtain the formation energies of the different interstitial configurations (BO: basal octahedral, BS: split dumbbells in the basal planes, BC: basal crowdion, O: octahedral, S: split dumbbells along the c-axis, C: crowdions, M: a configuration close to BO, and P2S: obtained by rotating the S configuration), as represented in Figure 1 [21]. The interstitial formation energy was calculated as:

(4) $$E_{int}^{f}=E_{int}-E_{per}-\mu ,$$

where *E*_int_ is the system energy with an interstitial, *E*_per_ is the energy of the perfect structure, and *µ* is the chemical potential of the added Zr interstitial atom. The supercell volume was fixed after the insertion of a Zr atom and only the atom positions were relaxed. The migration energies were also determined with the climbing image nudged elastic band method (CI-NEB) [32] with 11 intermediate images among the interstitial configurations.

## 3. Results and Discussion

### 3.1. Interstitial Diffusion in Pure Zr and Zr Alloys

**Figure 2:**
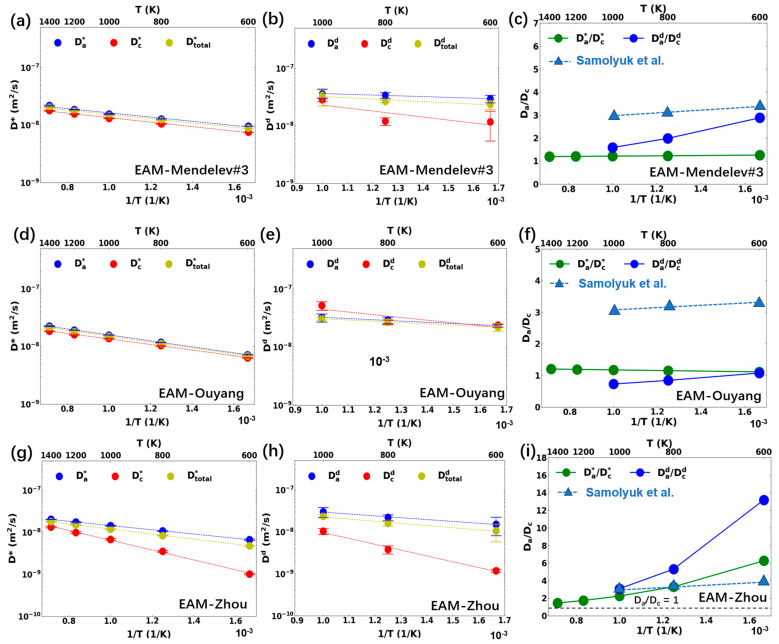
Diffusion behavior in pure Zr with (**a**–**c**) EAM-Mendelev#3, (**d**–**f**) EAM-Ouyang, and (**g**–**i**) EAM-Zhou potential: (**a**,**d**,**g**) tracer diffusion coefficients and (**b**,**e**,**h**) interstitial diffusion coefficients along different directions (**a**: basal plane, **c**: c-axis); (**c**,**f**,**i**) diffusion anisotropy parameters. The DFT results of anisotropy parameters from Samolyuk et al. [9] are marked as blue triangle.

Since interstitial anisotropic diffusion is one of the key factors in the evolution and anisotropic growth of irradiation-induced defects in Zr alloys, the interstitial diffusion coefficients and anisotropic diffusion behavior in the pure Zr and Zr alloys were studied using various MD potentials. Figure 2 presents the temperature-dependent tracer and interstitial diffusion coefficients, as well as the anisotropy parameters, along the basal plane and c-axis in the pure Zr for various interatomic potentials. Due to the correlation of defect motion between consecutive jumps, the tracer and interstitial diffusion coefficients were different, which also led to a difference in the anisotropic parameters between the atomic and defect diffusions. The anisotropy parameter derived from Samolyuk et al. [9] is also presented in Figure 2 for comparison. In their study, kinetic Monte Carlo (KMC) simulations of point defect jumps were performed according to the DFT migration energies among different SIA configurations. Recent work [24] suggests that interstitial migration may take place through a correlated motion between the interstitial atom and its neighboring atom, which was not considered in Ref. [9]. Nevertheless, the combined DFT and KMC results should provide a relatively accurate benchmark for comparison. As defect motion was tracked in the study of Samolyuk et al. [9], it should be mostly compared to $D_{a}^{d}/D_{c}^{d}$. The $D_{a}^{d}/D_{c}^{d}$ values at 1200 K and 1400 K were not calculated because the defect position could not be reliably identified due to the severe lattice vibration. Based on the tracer diffusion coefficients shown in Figure 2, the pre-exponential parameters and activation energies were also calculated, as presented in Table 1. The EAM-Mendelev#3 potential had the highest total diffusion coefficient for the studied temperature range, followed by EAM-Ouyang and then EAM-Zhou. The difference fell within the same order of magnitude, with the largest discrepancy being 50% between EAM-Mendelev#3 and EAM-Zhou at 600 K. The defect anisotropic parameters from EAM-Mendelev#3 were larger than unity, indicating a preference for basal diffusion. This preference became more pronounced at lower temperatures. The anisotropic parameters from EAM-Ouyang were close to one and increased with temperature. The results from these two potentials were both significantly lower than those obtained from the DFT+KMC method. The defect anisotropic parameters from EAM-Zhou exhibited a strong temperature dependence and were above the DFT+KMC results over the studied temperature range. The atomic anisotropic parameters from EAM-Zhou tended to have a more moderate temperature dependence and were lower than those of interstitial diffusion. The activation energies for the basal and c-axis directions were 0.10 eV and 0.23 eV, respectively, indicating a more pronounced reduction in the c-axis diffusion with temperature. Overall, significant differences in interstitial diffusion anisotropy were observed among the three potentials, in terms of both magnitude and temperature dependence.

**Figure 3:**
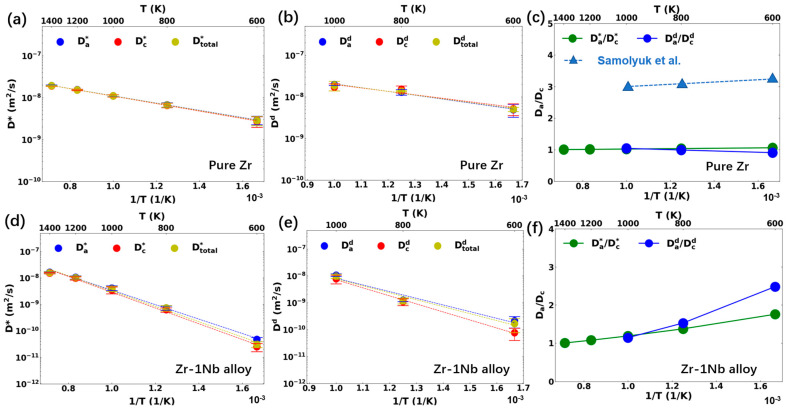
Diffusion behavior in (**a**–**c**) pure Zr and (**d**–**f**) Zr-1Nb alloy with the ADP-Starikov potential: (**a**,**d**) tracer diffusion coefficients and (**b**,**d**) interstitial diffusion coefficients along different directions (**a**: basal plane, **c**: c-axis); (**c**,**f**) diffusion anisotropy factors. The DFT results of anisotropy parameters from Samolyuk et al. [9] are marked as blue triangle.

The tracer and interstitial diffusion coefficients and anisotropy parameters of Zr and Zr-1Nb using the ADP-Starikov potential are presented in Figure 3. For pure Zr, the tracer diffusion coefficient exhibited an overall stronger temperature dependence compared to the aforementioned three EAM potentials, as evidenced by the higher activation energies (see Table 1). There was no noticeable diffusion anisotropy for the studied temperate range between 600 K and 1400 K, which was underestimated compared to the DFT+KMC results [9].

When 1.0 at. % Nb was added, there was a significant reduction in both the atomic and defect diffusion coefficients. It was found that the Zr-Nb interstitial tended to form and diffused slowly compared to the diffusion of the Zr interstitial or Zr-Zr dumbbell in pure Zr. The diffusion of each individual element will be further discussed in Section 3.3. The self-diffusion coefficient of the dilute Zr-1Nb alloy, which included the equilibrium interstitial concentration, was compared with the experimental results in the original study of ADP-Starikov, showing a good agreement in magnitude [22]. The inclusion of 1% of Nb significantly increased the total activation energy to 0.58 eV. Pronounced diffusion anisotropy was observed in Zr-1Nb, showing an increase in anisotropy as the temperature decreased. Despite the discrepancy in pure Zr diffusion anisotropy, the results here suggest that Nb interstitial migration is favored in the basal direction, especially at lower temperatures.

**Figure 4:**
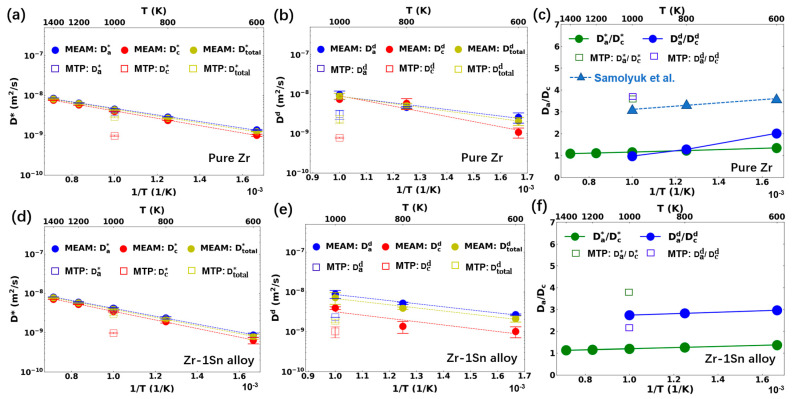
Diffusion behavior in (**a**–**c**) pure Zr and (**d**–**f**) Zr–1Sn alloy with MEAM-Mei and MTP-Mei potential: (**a**,**d**) tracer diffusion coefficients and (**b**,**d**) interstitial diffusion coefficients along different directions (**a**: basal plane, **c**: c-axis); (**c**,**f**) diffusion anisotropy factors. The DFT results of anisotropy parameters from Samolyuk et al. [9] are marked as blue triangle.

The diffusion behaviors of Zr and Zr-1Sn with the MEAM-Mei potential and MTP-Mei potential are presented in Figure 4. Compared to the four potentials shown in Figure 2 and Figure 3, the diffusion coefficients in the pure Zr from the MEAM-Mei potential were lower over the studied temperature range. The total activation energy was similar to that from ADP-Starikov (0.17 eV), but lower than the three EAM potentials. With the MTP-Mei potential, the diffusion simulation was only performed at 1000 K due to the slower computational efficiency of the machine learning potential. Based on the comparison at 1000 K, the MTP-Mei potential exhibited the lowest diffusion coefficient. Regarding the anisotropic parameter, it increased with a decrease in temperature for the MEAM-Mei potential, showing a similar trend to that observed in the DFT+KMC results (see Figure 4c). However, the proportion of basal diffusion was still underestimated. For instance, the defect anisotropic parameter $D_{a}^{d}/D_{c}^{d}$ was approximately 2 at 600 K, which is about 63% of the value from the DFT+KMC method. With the MEAM-MTP potential, the defect anisotropic parameter was close to the DFT+KMC result at 1000 K, showing that the basal diffusion was greatly favored.

When 1.0 at. % Sn was added into α-Zr, the atomic diffusion coefficient was not greatly affected (see Figure 4d). A slight increase in the pre-exponential factor and activation energy was observed for the MEAM-Mei potential. The diffusion of individual chemical species of Zr and Sn in Zr-1Sn will be discussed in Section 3.3. It is interesting to note that, while the atomic anisotropic parameter did not greatly differ from unity, the defect anisotropic parameter was high. The Sn atoms barely diffused in the Zr-1Sn structure, but they greatly suppressed interstitial diffusion along the c-axis direction with respect to the basal direction. This behavior was consistent for the three studied temperatures (see Figure 4e). The correlation in interstitial migration was significant in Zr-1Sn, resulting in a difference in the atomic and defect anisotropy. For the MTP-Mei potential, the diffusion coefficients in Zr-1Sn were similar to those of pure Zr. The anisotropic parameter exhibited a trend opposite to that observed with the MEAM-Mei, with a lower defect anisotropic parameter compared to the atomic anisotropic parameter. However, consistent with the result of MEAM-Mei, diffusion in the basal plane was greatly favored. According to the studied potentials, the addition of Sn did not induce significant variation in the total diffusion coefficient, but promoted the interstitial diffusion anisotropy.

### 3.2. Molecular Static Calculations of Interstitial Properties

The interstitial formation energies are presented in Table 2 for the studied potentials. The formation energies calculated from the original studies are also presented in italics. The interstitial positions of various configurations can be found in Ref. [21]. P2S’ is a configuration close to P2S, but differs in its exact interstitial orientation. First, most of our calculated values were in good agreement with those from the original studies. Some small discrepancies may have been due to the differences in the supercell size and the volume relaxation method. In our simulations, the dimensions of the 1008-atom supercell were fixed after the addition of the interstitial atom. The a/c ratio of the HCP structure has been known to also affect the energy and stability of interstitial configurations. However, there was a large discrepancy between our results and those from EAM-Ouyang for all configurations. We note that the same procedure was used in this work to calculate the formation energies of all the potentials.

**Figure 5:**
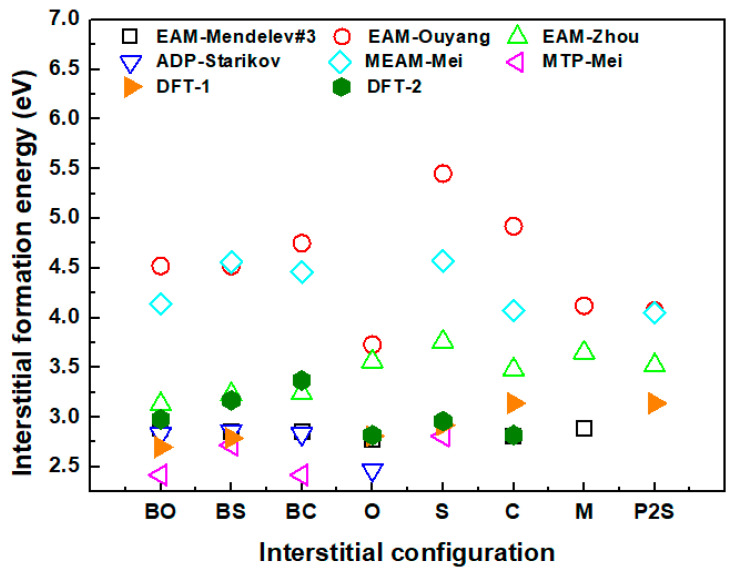
The interstitial formation energies of different interstitial configurations with the studied potentials and compared with DFT results [1,17].

Second, we noticed a large difference in the magnitude and the relative order of the interstitial formation energies among the different potentials (see Table 2 and Figure 5). The BO configuration is the most stable interstitial configuration according to DFT studies with large supercells [1,9,17]. However, only the BO configurations in EAM-Zhou and MTP-Mei had the lowest formation energy. We note that the BC configuration also had the same lowest formation energy as that of BO in the MTP-Mei. In the MEAM-Mei potential, although the BO structure was not the most stable structure, the formation energy was relatively low and was only 0.07 eV–0.09 eV higher than the most stable C and P2S structures. With EAM-Mendelev#3, EAM-Ouyang, and ADP-Starikov, the O configuration was the most stable structure. Next, according to DFT calculations, the BS and O configurations should have relatively low formation energies, differing by 0.1–0.2 eV from the BO configuration [1,17]. Meanwhile, other configurations should have higher formation energies. These two trends can hardly be fully satisfied by the studied DFT potentials; either the formation energies for the BS or O configuration were not sufficiently low, or other configurations also had low formation energies. Nevertheless, most potentials could maintain a fairly good consistency in relative energy for some of the interstitial structures. The EAM potential lacks angular dependence in its energy formulation. Therefore, it may be challenging to accurately represent all the interstitial configurations.

The migration energies among the different interstitial configurations are presented in Table 3. Migration paths involving the BO state are shown because BO is the most stable configuration. Some migration barriers related to the O configuration are also presented, because some potentials identified the O configuration as the most stable structure. For certain potentials, the studied migration had an intermediate stable state, which is presented in Table 3 as two sets of forward/backward migration barriers. The configurations labeled as M′, BS′, O′, and O″ are configurations close to M, BS, and O but differ slightly in their exact interstitial position and system energy. When the migration energy is shown as zero or close to zero, it indicates that the configuration is a metastable state along the migration path, or it has an extremely small migration barrier.

Table 3 shows that most migration barriers derived from the interatomic potentials differed significantly from those obtained via the DFT calculations. BO-BS jumps and BO-O jumps are two important pathways for basal and non-basal migrations, respectively [9]. However, no potential describes these two transitions accurately. The M configuration is close to BO, but is not exactly aligned in the basal plane, resulting in a small migration barrier in DFT calculations. For MEAM-Mei and MTP-Mei, the M configuration was spontaneously relaxed to BO without any migration barrier. A small BO-M barrier was observed only in EAM-Mendelev#3, whereas other potentials behaved differently between BO and M. P2S is a rotated S configuration and is relatively high in energy according to the DFT results. It is the local maximum along the BC’-S path [9]. The EAM-Mendelev#3, ADP-Starikov, and MTP-Mei reflected this unstable nature of P2S. The energy of P2S was relatively high but still lower than S in EAM-Zhou. For BO-S and O-M jumps, the EAM-Zhou potential had a similar energy landscape as the DFT calculations, only with some differences in numerical values. The other potentials exhibited either an unstable initial/final configuration or an intermediate stable state. O-BS migration involves two low-barrier jumps of O-M and M-BS, which was not accurately depicted by the studied potentials.

In general, migrations with low barriers are highly likely to occur during diffusion, and migrations with high barriers are less likely to take place and are greatly suppressed at low temperatures. For the EAM-Mendelev#3 potential, the most dominant migration jumps were BO-O and BO-BS with low energy barriers. Low barriers in both the forward and backward directions can lead to a high jump frequency. For the EAM-Ouyang potential, the migration barriers between the most stable O configuration and other configurations were large. The overall low activation energy observed during diffusion (see Table 1) suggested that other low-energy transition states, apart from those deemed dominant by the DFT calculations, may exist for this potential. For the EAM-Zhou potential, the basal diffusion of BO-BS-BO migration was highly likely; however, the migration pathways along the c-axis had high migration energies, which can explain the strong diffusion anisotropy along the basal direction for this potential. For the ADP- Starikov potential, the O configuration was the most stable, and multiple configurations (C, M, and P2S) relaxed to O spontaneously. Its weak diffusion anisotropy can be associated with its lower probability of basal diffusion. Although BO-BS transition has low migration barriers, it is less likely to transform to BO and BS due to their higher formation energies. For the MEAM-Mei potential, BO had the lowest formation energy and O could also relax to the BO configuration. BO-BS-BO migration is an important basal diffusion pathway, where BS is a saddle point between BO and BO. Contrary to the BO-O migration predicted by the DFT method, the BO-P2S and BO-S jumps contributed to the non-basal diffusion for this potential. For the MTP-Mei potential, BO and BC were the most stable interstitial configurations, and O could also automatically transform to the BO configuration. BO-BS migration has relatively low barriers, which led to the preferred basal diffusion with this potential. Although other probable transition states that are not listed in Table 3 can also exist, the results here show that these potentials exhibit significantly different diffusion dynamics. While the molecular dynamics results may appear similar among some of the studied potentials, the underlying migration routes and jump correlations could be dramatically different.

### 3.3. Solute Atom Diffusion in Zr Alloys

**Figure 6:**
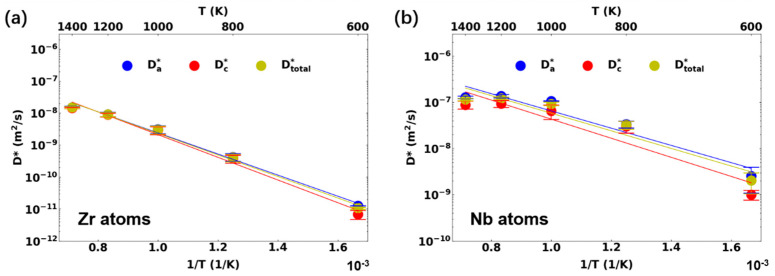
Elemental diffusion behavior in Zr-1Nb alloy with the ADP-Starikov potential: (**a**) Zr atoms; (**b**) Nb atoms.

**Figure 7:**
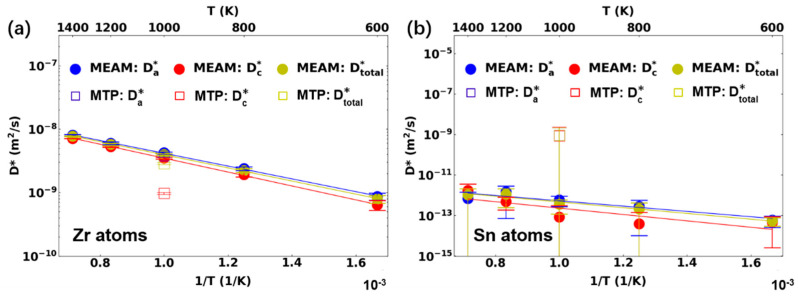
Elemental diffusion behavior in Zr-1Sn alloy with the MEAM-Mei potential: (**a**) Zr atoms; (**b**) Sn atoms.

The diffusion coefficients of Zr and Nb in Zr-1Nb using the ADP-Starikov potential, as well as those of Zr and Sn in Zr-1Sn using the MEAM-Mei potential, are shown in Figure 6 and Figure 7, respectively. The tracer diffusion coefficient was calculated with Equation (1), which was normalized by the interstitial concentration in the supercell. The Nb diffusion coefficient was one to two orders of magnitude larger than that of the Zr atoms in Zr-1Nb, meaning that every Nb atom diffused faster than the Zr atoms on average. When comparing the Zr diffusion in pure Zr (see Figure 3a), the Zr atoms in Zr-1Nb diffused slower, indicating that 1 at. % of Nb inhibited the Zr diffusion, accompanied by the significant diffusion of Nb. This is consistent with previous DFT calculations, which show that the Nb interstitial at the O site is ~0.35 eV lower than the most stable Zr self-interstitial atom [11]. The faster diffusion of Nb was also qualitatively consistent with diffusion experiments [33,34]. It is noticed from Figure 6b that Nb diffusion deviated significantly from the Arrhenius relationship and had a complicated temperature dependence. The Nb interstitial resulted in enhanced atomic diffusion anisotropy along the basal direction.

In the Zr-1Sn alloy, the Sn atoms exhibited extremely low diffusion coefficients with the MEAM-Mei potential, which were about four orders of magnitude lower than those of Zr. The lower Sn mobility resulted in the large uncertainty shown in Figure 7b. Based on DFT calculations, the Zr-Sn interstitial had low formation energies, with the most stable configuration being ~1 eV lower than the most stable Zr interstitial atom. This indicated that a Zr-Sn interstitial should be highly likely to form during diffusion [11]. This seems contradictory to the results from the studied potential. The Zr-Sn cross-interactions mostly took into account the properties of the various Zr-Sn intermetallic compounds. Therefore, the Sn-related defect properties in Zr solid solution may not be accurately described. Nevertheless, it is worth noting that, with MEAM-Mei, the addition of 1 at% Sn changes the chemical environment of Zr interstitial atoms, resulting in a stronger interstitial diffusion anisotropy with respect to pure Zr. This effect highlights the importance of dilute solute atoms on Zr diffusion. With the MTP-Mei potential, the Zr diffusion coefficient in Zr-1Sn was close to that of MEAM-Mei along the basal plane, but the diffusion along the c-axis was greatly suppressed. The Sn atoms in Zr-1Sn diffused slightly slower than the Zr atoms in Zr-1Sn, but much faster than the Sn atoms in the MEAM-Mei potential. In addition, Sn diffusion did not exhibit any diffusion anisotropy. However, the addition of Sn changed the interstitial diffusion anisotropy of the overall alloy (see Figure 4), suggesting that solute atoms have a profound impact on interstitial migration dynamics. Determining the actual behavior of Sn solutes in Zr requires the consideration of different migration pathways and needs to be validated through DFT calculations. This further indicates that the accuracy of interstitial diffusion heavily depends on the interatomic potential, including both its form and the properties considered in its fitting.

## 4. Conclusions

In this study, we studied the diffusion behavior and energy properties of various interstitial configurations in pure Zr and Zr alloys. From the results of this study, the following conclusions can be drawn:

1. Significant differences in the diffusion coefficient and diffusion anisotropy were observed among different interatomic potentials. For pure Zr, the EAM-Mendelev#3 and EAM-Zhou potentials indicated that the interstitials exhibited an anisotropic diffusion trend, with the degree of anisotropy increasing with a decreasing temperature. Conversely, the EAM-Ouyang potential function exhibited weak anisotropic diffusion. For Zr-1Nb, the ADP-Starikov potential predicted that the addition of the solute element Nb reduced the interstitial diffusion compared to pure Zr. It also exhibited clear anisotropic behavior during defect diffusion, with an increased degree of anisotropy with a decreasing temperature. For Zr-1Sn, MEAM-Mei and MTP-Mei predicted that the addition of the solute element Sn would not lead to significant changes in the overall interstitial diffusion; however, the degree of defect diffusion anisotropy was increased with the addition of Sn.
2. Most of the potentials showed reasonable agreement with the DFT results regarding the interstitial static energy calculations, but differences were observed among these potentials in terms of the magnitude of the interstitial formation energies and their relative orders. According to the DFT studies, the BO configuration was the most stable interstitial configuration and should be reproduced by classical potentials for accurately modeling interstitial diffusion. The analysis of the migration pathways showed that different potentials exhibited pronounced differences in the migration barriers among various interstitial configurations.
3. The solute atoms of Nb and Sn can have significant impacts on the interstitial diffusivity or diffusion anisotropy. Solute atoms can either participate in interstitial diffusion themselves or influence Zr diffusion due to changes in the local chemical environment.

Overall, this study underscores the complexity in accurately modeling the interstitial behavior in α-Zr and Zr alloys, emphasizing the necessity of validating and selecting suitable potentials and understanding the influence of alloy elements in interstitial diffusion. In future research, it would be beneficial to explore the diffusion behavior of vacancy defects and defect clusters in Zr alloys. This also includes considering a broader range of properties and factors in the fitting and selection of potential functions.

## Figures and Tables

**Table 1:** The pre-exponential parameters and activation energies from Arrhenius fitting of tracer diffusion coefficients derived from different potentials.

Material	Potential	D_0, basal_ (m^2^/s)	E_a, basal_ (eV)	D_0, c-axis_ (m^2^/s)	E_a, c-axis_ (eV)	D_0, total_ (m^2^/s)	E_a, total_ (eV)
Zr	EAM-Mendelev#3	3.78 × 10^−8^	0.07	3.29 × 10^−8^	0.08	3.61 × 10^−8^	0.07
Zr	EAM-Ouyang	5.21 × 10^−8^	0.10	4.08 × 10^−8^	0.10	4.83 × 10^−8^	0.10
Zr	EAM-Zhou	4.60 × 10^−8^	0.10	9.60 × 10^−8^	0.23	4.74 × 10^−8^	0.12
Zr	ADP-Starikov	8.09 × 10^−8^	0.17	8.42 × 10^−8^	0.18	8.19 × 10^−8^	0.17
Zr	MEAM-Mei	3.13 × 10^−8^	0.16	3.41 × 10^−8^	0.18	3.20 × 10^−8^	0.17
Zr-1Nb	ADP-Starikov	1.83 × 10^−6^	0.54	2.78 × 10^−6^	0.59	2.53 × 10^−6^	0.58
Zr-1Sn	MEAM-Mei	4.19 × 10^−8^	0.20	4.30 × 10^−8^	0.22	4.21 × 10^−8^	0.21

**Table 2:** Formation energies of various SIA configurations (in eV) by different empirical potentials. The values from the original classical potentials are presented in italics in the parenthesis. The unstable configurations are labeled with “U” with the stable configuration shown in the parenthesis.

Empirical Potential	BO	BS	BC	O	S	C	M	P2S
EAM-Mendelev#3 [18]	2.89	2.86	2.86	2.78	U (O)	2.81	2.89	U (O)
	*(2.90)*	*-*	*-*	*(2.88)*	*-*	-	-	-
EAM- Ouyang [19]	4.52	4.52	4.75	3.73	5.45	4.92	4.12	4.07
	*(3.30)*	*(3.10)*	*(3.08)*	*(3.04)*	*(3.74)*	*(3.23)*	*-*	*-*
EAM- Zhou [21]	3.13	3.23	3.24	3.56	3.76	3.48	3.65	3.52
	*(3.18)*	*(3.31)*	-	*(3.52)*	*(3.42)*	*(3.32)*	-	*(3.26)*
ADP- Starikov [22]	2.83 *(2.88)*	2.86 *(2.91)*	2.83 *(2.88)*	2.47 *(2.49)*	U (P2S’) *(3.41)*	U (O) *(2.49)*	U (O) -	U (O) *-*
MEAM- Mei [23]	4.14	4.56	4.46	U (BO)	4.57	4.07	U (BO)	4.05
	*(4.09)*	*(4.51)*	*(4.09)*	*(5.56)*	*(4.62)*	*(4.05)*	*-*	*-*
MTP- Mei [23]	2.42	2.72	2.42	U (BO)	2.81	U (S)	U (BO)	U (S)
	*(2.40)*	*(2.71)*	*(2.40)*	*(3.24)*	*(2.78)*	*(2.40)*	*-*	*-*
DFT-1 [1]	2.70	2.79	-	2.81	2.92	3.14	-	3.14
DFT-2 [17]	2.98	3.17	3.37	2.82	2.96	2.82	-	-

**Table 3:** Migration energies of different migration paths for SIAs in α-Zr (in eV) by different empirical potentials. For unstable initial or final configurations, they are labeled with “U”. For migration paths with an intermediate state, two sets of migration barriers are provided with the intermediate configuration labeled in parenthesis.

Empirical Potential	Direction	BO-BS	BO-O	BO-M	BO-P2S	BO-S	O-BS	O-C	O-M
EAM- Mendelev#3 [13]	Forward *Backward*	0.000 *0.025*	0.005 *0.029*	0.000 *0.027*	U	U	0.698 *0.617*	0.382 *0.357*	0.114 *0.001*
	Forward *Backward*	0.029 *0.113*		0.031 *0.000*					
		(M)		(M′)					
EAM- Ouyang [14]	Forward	0.012	0.067	0.005	0.185	1.172	0.794	1.195	0.000
	*Backward*	*0.574*	*0.859*	*0.639*	*0.593*	*0.247*	*0.000*	*0.011*	*0.213*
	Forward *Backward*	0.564 *0.000*		0.270 *0.041*	0.070 *0.111*				0.597 *0.000*
		(BS′)		(M′)	(O′)				*(O*″)
EAM- Zhou [15]	Forward	0.154	0.582	0.523	0.496	0.825	0.374	0.659	0.080
	*Backward*	*0.056*	*0.140*	*0.000*	*0.102*	*0.197*	*0.717*	*0.751*	*0.000*
ADP- Starikov [16]	Forward	0.145	0.009	U	U	U	0.768	U	U
	*Backward*	*0.112*	*0.363*				*0.381*		
MEAM- Mei [17]	Forward	0.427	U	U	0.159	0.384	U	U	U
	*Backward*	*0.000*			*0.253*	*0.498*			
	Forward					0.537			
	*Backward*					*0.000*			
						(O′)			
MTP- Mei [17]	Forward	0.303	U	U	U	0.311	U	U	U
	*Backward*	*0.003*				*0.298*			
	Forward					0.613			
	*Backward*					*0.238*			
						(BO′)			
DFT [22]	Forward	0.254	0.269	0.073		0.526	0.115		0.115
	*Backward*	*0.124*	*0.123*	*0.008*		*0.275*	*0.196*		0.196
	Forward *Backward*						0.150 *0.086*		
							(M)		

## Data Availability

The original contributions presented in the study are included in the article, further inquiries can be directed to the corresponding authors.
